# Cement Removal from the Femur Using the ROBODOC System in Revision Total Hip Arthroplasty

**DOI:** 10.1155/2013/347358

**Published:** 2013-10-22

**Authors:** Mitsuyoshi Yamamura, Nobuo Nakamura, Hidenobu Miki, Takashi Nishii, Nobuhiko Sugano

**Affiliations:** ^1^Center of Arthroplasty, Kyowakai Hospital, 1-24-1 Kishibekita, Suita, Osaka 564-0001, Japan; ^2^Department of Orthopaedic Surgery, Osaka University Graduate School of Medicine, 2-2 Yamadaoka, Suita, Osaka 565-0871, Japan; ^3^Department of Orthopaedic Medical Engineering, Osaka University Graduate School of Medicine, 2-2 Yamadaoka, Suita, Osaka 565-0871, Japan

## Abstract

*Introduction*. The perforation and fracture of the femur during the removal of bone cement in revision total hip arthroplasty (THA) are serious complications. The ROBODOC system has been designed to selectively remove bone cement from the femoral canal, but results have not been reported yet. The purpose of our study was to evaluate the clinical and radiographic results of revision THA using the ROBODOC system for cement removal. *Materials and Methods*. The subjects comprised 19 patients who underwent revision THA using the ROBODOC system. The minimum duration of follow-up was 76 months (median, 109 months; range, 76–150 months). The extent of remaining bone cement on postoperative radiography, timing of weight bearing, and the complications were evaluated. *Results*. The mean Merle d'Aubigne and Postel score increased from 10 points preoperatively to 14 points by final follow-up. Bone cement was completely removed in all cases. Full weight bearing was possible within 1 week after surgery in 9 of the 19 cases and within 2 months in all remaining cases. No instances of perforation or fracture of the femur were encountered. *Conclusions*. Bone cement could be safely removed using the ROBODOC system, and no serious complications occurred. Full weight bearing was achieved early in the postoperative course because of circumferential preservation of the femoral cortex.

## 1. Introduction

The perforation and fracture of the femur during the removal of bone cement in revision total hip arthroplasty (THA) are serious complications that considerably affect the postoperative protocols and clinical results [1]. With the increasing frequency of revision THA, the incidence of intraoperative femoral fracture has increased recently [2, 3]. To prevent the perforation and fracture of the femur, several instruments and procedures have been developed especially for bone cement removal. However, sufficient results have not been achieved yet in the clinical setting [4–7]. Extended trochanteric osteotomy was introduced for difficult situations in revision THA [8–11], but good results have not necessarily been obtained with the procedure in terms of intraoperative femoral fracture [8, 10, 12].

Since 1992, a computer-assisted surgical system called ROBODOC (Integrated Surgical Systems, Davis, CA) has been used in clinical settings and is highly regarded for the accuracy of the surgical process [13–15]. After making system improvements, the ROBODOC system received 510(k) clearance from the US Food and Drug Administration in 2008. Using ROBODOC, the rate of intraoperative femoral fissures was significantly lower than that of the hand rasping conventional THA [16]. This system can also selectively remove bone cement from the femoral canal in revision THA [13]. However, results have not been reported yet.

The purpose of our study was to evaluate the clinical and radiographic results of revision THA using the ROBODOC system. Our research questions were as follows: (1) Did the system contribute to a reduced rate of intraoperative complications such as femoral fracture? (2) Did the use of the system affect the postoperative rehabilitation protocol with regard to weight bearing? (3) Was bone cement completely removed from the femur? 

## 2. Materials and Methods

We reviewed the medical records and radiographs of the studied subjects after the approval of this study by the institutional review board committee. The subjects comprised 19 patients (17 women, 2 men) for whom bone cement of the femoral canal was removed using the ROBODOC system in revision THA, between 2000 and 2006. All patients provided informed consent for participation before surgery, and the procedure was approved by the institutional review board committee. The mean patient age at the time of surgery was 70 years (range, 51–85 years). Cemented femoral component had been implanted in all patients. The primary diagnosis was osteoarthritis in 14 hips, femoral neck fracture in 4 hips, and rheumatoid arthritis in 1 hip. The reason for revision was aseptic loosening in 17 hips, septic loosening in 1 hip (infection was completely cleared up at the time of surgery), and central migration of the bipolar head in 1 hip. The minimum duration of follow-up was 76 months (median, 109 months; range, 76–150 months).

**Figure 1:**
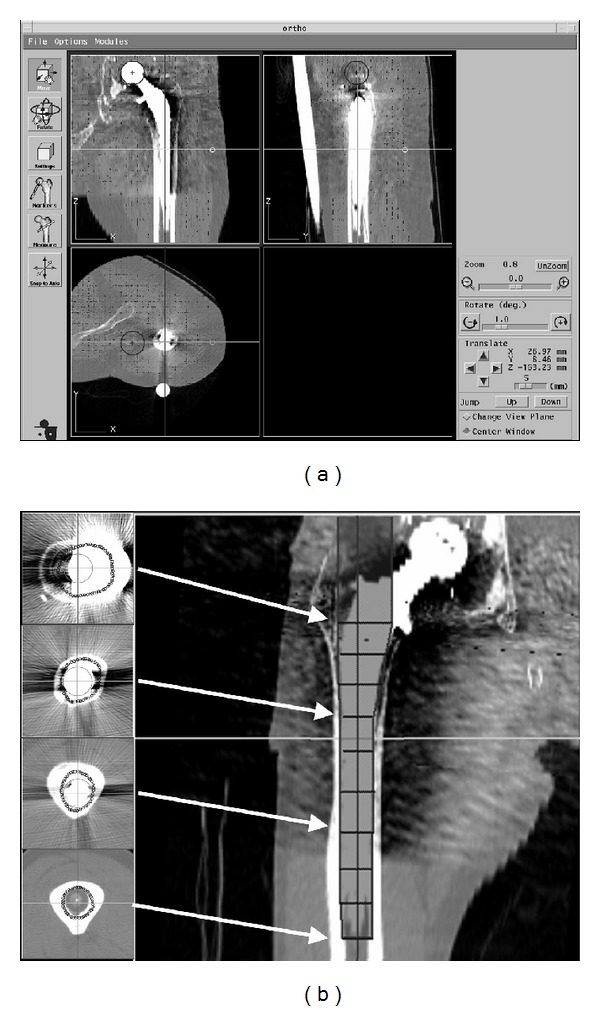
(a) Multiplanar reconstruction of the proximal femur on ORTHODOC. A minimum of 8 cross sections are defined on a coronal view. A perimeter around the bone is demarcated on each section. (b) A cutting path (hatched area) is automatically created.

Prior to the index surgery, 2 locater pins were implanted into the greater trochanter and lateral condyle of the affected femur under local anesthesia. Computed tomography (CT) (General Electric, Waukesha, WI) was then performed in accordance with the protocol specified by the manufacturer (slice thickness, 1 mm; scan interval, 1–6 mm; field of view, 200 mm; total slices, <200). CT data were imported into a preoperative planning workstation (ORTHODOC; Integrated Surgical Systems, Davis, CA) that displayed a 3-dimensional image of the femur (Figure 1(a)). The long axis of the femur was aligned. At least 8 cross sections were defined, and the surgeon demarcated a perimeter around the bone cement in the axial views of the femur. From these data, the ORTHODOC program automatically created a 3-dimensional cutting path for cement removal (Figure 1(b)). At this time, the surgeon could check and modify the cutting path. These preoperative planning data were recorded on a compact disc (CD). Before each surgical procedure, the surgeon loaded the data for that patient from this CD into the ROBODOC system and performed a startup self-diagnosis of the robot.

**Figure 2:**
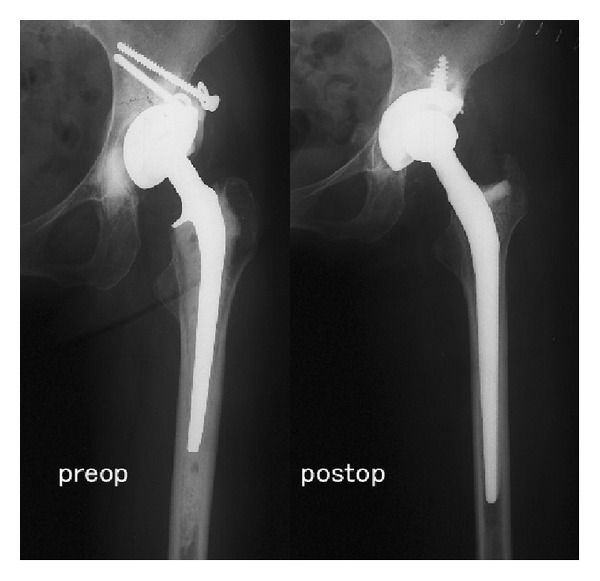
A 54-year-old woman underwent primary THA for osteoarthritis of the hip. Eleven years later, revision THA was performed using the ROBODOC system due to aseptic loosening. Full weight bearing was possible within 1 week after surgery. At the latest follow-up, 9 years postoperatively, no clinical or radiographic problems were identified.

During the operation, the femur was exposed through a posterolateral approach in all cases, and the femoral component was removed using a conventional procedure. After the patient's leg was fixed to ROBODOC and to the surgical table, registration was performed using the 2 locater pins. After the gluteus medius muscle was firmly retracted, ROBODOC milled the femoral canal to remove the bone cement. Finally, the surgeon performed manual reaming of the femoral canal and a long-straight-tapered cementless stem (Wagner, Zimmer, Warsaw, USA) was inserted (Figure 2).

Clinical and radiographic evaluations were performed on the day of operation and 6 weeks and 3, 6, and 12 months postoperatively, then annually thereafter. No patients were excluded or lost to followup. Clinical results were measured using Merle d'Aubigne and Postel score [17]. Each patient was questioned on each visit, regarding the presence of thigh pain, which was considered as a complication related to the femoral component. The time at which full weight bearing was resumed was recorded. The extent of remaining bone cement was assessed on postoperative anteroposterior and lateral radiographs of the femur, taken immediately after surgery. A stem was considered unstable when progressive subsidence >3 mm, any change in position, or a continuous radiolucent line wider than 2 mm was seen [18].

## 3. Results

The patients' demographic and operative data are provided in Table 1. The mean operation time was 267 min (range, 180–420 min). The mean robotic milling time was 34 min (range, 17–51 min). The mean blood loss was 1236 g (range, 450–3000 g). The mean clinical score increased from 10 points (range, 6–12 points) preoperatively to 14 points (range, 9–17 points) at final follow-up. No instances of the perforation or fracture of the femur were seen during surgery or follow-up. No patients in this series displayed nerve palsy or infection, including locater pin cite. The only patient who complained of thigh pain displayed 3 mm of stem subsidence immediately after surgery. However, symptoms disappeared when subsidence stopped 6 months after surgery. Further revision was required in 2 cases for acetabular loosening.

Of the 19 cases, full weight bearing was possible within 1 week in 9 cases. In other 9 cases, because bone grafting was performed to correct an acetabular bone defect, the patients were forced to delay full weight bearing due to the unreliable stability of the acetabular cup. The remaining patient was the first case in this study, and we were overly careful not to allow full weight bearing too early. However, full weight-bearing was achieved by all 19 patients within 2 months postoperatively, and they all became able to walk with a single cane.

Radiographically, bone cement was completely removed in all cases. Stem subsidence was seen in 2 cases, including the one mentioned previously. In the other case, subsidence progressed to 2 cm because of an undersized stem. Dislocation was seen only in this patient and was successfully treated using an abduction brace for three months after close reduction. As of final follow-up, all stems were considered to be bone stable.

## 4. Discussion

The results of revision THA using the ROBODOC system have not previously been reported. The purpose of this study was to evaluate the clinical and radiographic results for the ROBODOC system. In particular, we focused on the presence of intraoperative femoral fracture, the timing of full weight bearing, and the extent of remaining bone cement.

Several limitations in this study warrant consideration. First, the study design was retrospective, and we had no control group. Second, the number of cases was small, so the efficacy of this system needs to be confirmed in a larger number of cases. In addition, this system is strictly designed for the removal of bone cement and cannot be used for the removal of the stem itself. The primary disadvantages of this system are the need to implant locater pins before the revision surgery and the cost of the equipment.

The rate of the intraoperative fractures of the femur in revision THA ranges widely from 2.3% to 50% [19–23]. To remove the intramedullary bone cement safely, many new instruments and procedures, such as a ballistically driven chiseling system [7], a water jet [6], and high-energy shock waves [24], have been introduced, but clinical results have not yet been reported. Although an ultrasonic device presented some good clinical results [4, 5], Gardiner et al. reported complications related to the use of the device, such as superficial bone burns (9%) and bone perforation (3.3%) [5]. In addition, cases of radial nerve palsy and pathological humeral fracture reportedly developed after ultrasonic cement removal from the humerus [25]. Extended trochanteric osteotomies have been recommended to facilitate femoral component removal, femoral cement removal, and acetabular exposure in cases of difficult revision THA [8–11]. However, Noble et al. reported in an in vitro cadaveric study that extended trochanteric osteotomy reduced the torsional strength of the femur by 73%, even when the osteotomy fragment was repaired [26]. Moreover, Busch et al. postulated in a series of 219 revision procedures that the use of extended trochanteric osteotomy would represent a risk factor for stem fracture after revision surgery due to the poor proximal femoral bone support [19]. Cement-in-cement techniques have been introduced to reduce intraoperative complications in cases of well-fixed cemented stem revisions. Some good midterm results have been reported, but the perforation and fracture of the femur could not be completely avoided (1.5–20.4%) [27–29]. The ROBODOC system has been used without femoral fracture in a total of 900 cases of primary THA, clearly establishing the safety of this method [13]. Of note is the fact that no perforations or fractures of the femur occurred in our series. The ROBODOC system has an advantage over other new procedures, because the cutting area can be assessed 3-dimensionally before surgery and reproduced reliably during the operation.

The ROBODOC system allows early full weight bearing because of the circumferential preservation of the femoral cortex, which may help reduce the hospital stay and expenses. Among our 19 cases, full weight bearing was possible within 1 week after surgery in 9 cases and in all remaining patients within 2 months. The use of the extended trochanteric osteotomy reduces the rates of nonunion and migration of the osteotomy site (0–2%) [9–11]. However, to prevent these events, postoperative rehabilitation protocols must be restrictive. Brace wear and partial weight bearing may be continued for about 8–12 weeks before full weight bearing is allowed [9, 11].

Removing all cement from the femoral canal without using a cortical window or osteotomy is challenging. Schurman reported that in 12 of 15 cases, cement mantles were completely removed using the segmental cement extraction system. However, 2 cases showed retained cement along the medial wall of the femur, and the plug could not be extracted using this system in 1 case [30]. Jingushi et al. reported remaining parts of cement in the canal in postoperative radiography in 65% patients (13/20) using standard instruments for revision arthroplasty [31]. The clinical reports of the ultrasonic device did not refer to whether bone cement was completely removed [4, 5]. In our study, bone cement was completely removed without osteotomy in all 19 cases, thanks to the 3-dimensional assessment of the cutting area and the reproducibility of the ROBODOC system.

Nogler et al. pointed out the risk of heat injury during the ROBODOC milling process of cement removal, if cooling facilities were insufficient [32]. Although heat generation may cause soft tissue damage and bone necrosis, no nerve palsy or pathological fracture was encountered in our series, and bone ongrowth fixation between stem and femur was achieved in all cases. This result indicates that the intramedullary irrigation system functioned well for cooling, and bone around the stem remained viable.

In revision THA using the ROBODOC system, bone cement could be safely removed without the perforation or fracture of the femur, and full weight bearing was achieved early in the postoperative course due to the circumferential preservation of the femoral cortex.

## Figures and Tables

**Table 1:** The patients demographic and operative data.

Case	Age (years)	Sex	Primary diagnosis	Reason for revision	Operation time (min)	Blood loss (g)	FWB (weeks)	Stem subsidence
1	76	M	FNF	Aseptic loosening	210	600	9	−
2	72	F	OA	Aseptic loosening	225	1600	1	−
3	69	F	OA	Aseptic loosening	250	450	5	−
4	63	F	RA	Aseptic loosening	215	1100	1	−
5	51	F	OA	Aseptic loosening	180	960	4	+
6	70	F	OA	Aseptic loosening	225	550	5	−
7	78	F	OA	Aseptic loosening	258	1000	1	−
8	73	F	OA	Aseptic loosening	285	1100	9	−
9	71	M	FNF	Septic loosening	251	3000	1	+
10	74	F	OA	Aseptic loosening	420	1350	9	−
11	77	F	FNF	Aseptic loosening	300	700	8	−
12	54	F	OA	Aseptic loosening	285	570	1	−
13	64	F	OA	Aseptic loosening	405	1450	9	−
14	75	F	OA	Aseptic loosening	335	1750	1	−
15	82	F	OA	Aseptic loosening	205	1100	1	−
16	59	F	OA	Aseptic loosening	275	2100	1	−
17	80	F	OA	Aseptic loosening	212	1500	1	−
18	75	F	OA	Aseptic loosening	260	1000	1	−
19	72	F	FNF	Bipolar head migration	290	1600	1	−

Abbreviations: FWB: full weight bearing; FNF: femoral neck fracture; OA: osteoarthritis; RA: rheumatoid arthritis.
